# Climate action requires new accounting guidance and governance frameworks to manage carbon in shelf seas

**DOI:** 10.1038/s41467-020-18242-w

**Published:** 2020-09-15

**Authors:** Tiziana Luisetti, Silvia Ferrini, Gaetano Grilli, Timothy D. Jickells, Hilary Kennedy, Silke Kröger, Irene Lorenzoni, Ben Milligan, Johan van der Molen, Ruth Parker, Tim Pryce, R. Kerry Turner, Emmanouil Tyllianakis

**Affiliations:** 1 Centre for Environment, Fisheries, and Aquaculture Science (Cefas), Pakefield Road, Lowestoft, Suffolk NR33 0HT UK; 2 CSERGE, School of Environmental Sciences, University of East Anglia, Norwich Research Park, Norwich, NR4 7TJ UK; 3 School of Environmental Sciences, University of East Anglia, Norwich Research Park, Norwich, NR4 7TJ UK; 4 Bangor University, School of Ocean Sciences, Askew Street, Menai Bridge, Anglesey, LL59 5AB UK; 5 University of New South Wales, Faculty of Law, The Law Building, UNSW, Sydney, NSW 2052 Australia; 6 NIOZ Royal Netherlands Institute for Sea Research and Utrecht University, P.O. Box 59, 1790 AB Den Burg, Texel Netherlands; 7 Carbon Trust, 4th Floor, Dorset House, 27-45 Stamford Street, London, SE1 9NT UK; 8 Present Address: CSERGE, School of Environmental Sciences, University of East Anglia Norwich Research Park, Norwich, NR4 7TJ UK; 9 Present Address: Sustainability Research Institute, University of Leeds, Leeds, LS2 9JT UK

**Keywords:** Carbon cycle, Climate-change policy, Economics

## Abstract

Accounting guidelines exist for the recording of carbon flows in terrestrial and coastal ecosystems. Shelf sea sediments, while considered an important carbon store, have yet to receive comparable scrutiny. Here, we explore whether effective management of carbon stocks accumulating in shelf seas could contribute towards a nation’s greenhouse gas emissions reduction targets. We review the complexities of carbon transport and fate in shelf seas, and the geopolitical challenges of carbon accounting in climate governance because of the transboundary nature of carbon flows in the marine environment. New international accounting guidance and governance frameworks are needed to prompt climate action.

## Abstract (web summary)

Accounting guidelines exist for carbon flows in terrestrial and coastal ecosystems, but not shelf sea sediments. In this Review, the authors explore whether effective management of carbon stocks accumulating in shelf seas could contribute to a nation’s greenhouse gas emissions reduction targets.

## Introduction

Integration of natural capital into physical and economic accounts is being examined by many countries worldwide^1^. An important aim of such integrated accounts is to support the implementation of the UN Sustainable Development Goals (SDGs) and multilateral environmental agreements such as the UN Framework Convention on Climate Change (UNFCCC). Carbon sequestration, commonly defined in the natural sciences literature as the processes of carbon capture and storage, is an important element of such accounts because it may contribute towards a nations’ greenhouse gas emissions reduction contributions within the 2015 Paris Agreement and previous obligations under the UNFCCC Article 4 (Parties commitments to mitigate climate change)^2^.

Vegetated terrestrial systems such as forests sequester, capture and store carbon in their biomass and the soil beneath them. A similar process occurs in the marine environment where vegetated marine systems, such as salt marshes, mangroves and seagrass meadows, capture and store carbon. Not all carbon fixed by these systems (i.e. turned into biomass) becomes stored in the soil where it was produced; a fraction is eventually transported and stored (i.e. buried) in coastal or offshore shelf sea sediments. Shelf sea sediments not only store detritus generated by terrestrial and coastal vegetation, but also carbon inputs generated from phytoplankton productivity and other carbon sources (e.g. macroalgae) throughout the shelf seas and adjacent ocean environment. However, we still do not know what fraction of the accumulating organic matter, herewith termed as particulate organic carbon (POC), is derived from each of the potential sources listed above. Wide regional variations in the composition of the accumulating sediment are to be expected due to multiple and divergent organic matter sources^3–5^. Information is lacking also on the amount of organic carbon produced that becomes ultimately buried in shelf sea sediments.

Terrestrial vegetated ecosystems, including coastal ecosystems, which are classified as managed lands and which sequester carbon, are recognised by the UNFCCC through various governance mechanisms^6^. Marine ecosystems, in contrast, are less well represented and managed^7^. General obligations stated in Article 4 of the UNFCCC require all Parties to compile inventories of emissions by sources and removals by sinks based on specific Intergovernmental Panel on Climate Change (IPCC) guidelines. Given the sediments’ organic carbon density (8.8 mgC cm^−3^) and the large area of shelf seas (Table 1), which is ~7% of the global marine area^8,9^, and considering that they are a potentially manageable carbon store, we suggest that carbon sequestration in shelf sea sediments should be considered within the scope of both IPCC inventory and environmental–economic accounting methodologies. Proper management of the carbon currently sequestered into these invisible stores, in fact, may play a significant role in mitigating climate change. We consider the opportunities, challenges and benefits of the preservation of shelf sea sedimentary carbon as a contribution to climate change mitigation by considering the following critical questions: How is ownership of carbon stocks in shelf sea sediments distributed? How should these carbon stocks be measured and registered in national carbon accounts? What arrangements can be made to ensure the conservation of the carbon stocks?

**Table 1:** Global estimates of carbon burial in individual ecosystems defined by their vegetation, and/or geomorphological characteristics in coastal wetlands, shelf sediments and tropical forests; illustrating that on a global scale, the carbon sink in shelf systems is comparable to that in tropical forests.

Habitat	Area 10^6^ ha	Org C burial 10^6^ gC ha^−1^ yr^−1^	Global C burial Tg C yr^−1^
Mangrove	13.7–15.2^21^	1.62^49^	22.2–24.8
Saltmarsh	2.2–40^21^	0.91^49^	2.2–36
Seagrass	17.7–60^21^	0.43^49^	7.6–25.8
Shelf^a^	2700^98^	0.17^98^	45.2^98^–135.6^99^
Tropical forests	1962^100^	0.04^100^	78.5^100^

Most of that shelf carbon is buried within ecosystems defined by their geomorphological, rather than biological, features. While mangroves, saltmarshes and seagrass meadows are important sinks on a per unit area basis, their extent is relatively small, but significant, as they are vulnerable habitats to human disturbance. Note that carbon deposition in shelf sediments (<200-m depth) is roughly equivalent to that in the deep ocean >200-m depth^3^, and potentially vulnerable to human activities.

^a^The depositional areas on the shelf varies with their geographic setting. The range given for global C burial represents estimates for a depositional areal extent of muds between 10^98^ and 30%^99^ of the total shelf area reported in column two.

**Fig. 1:**
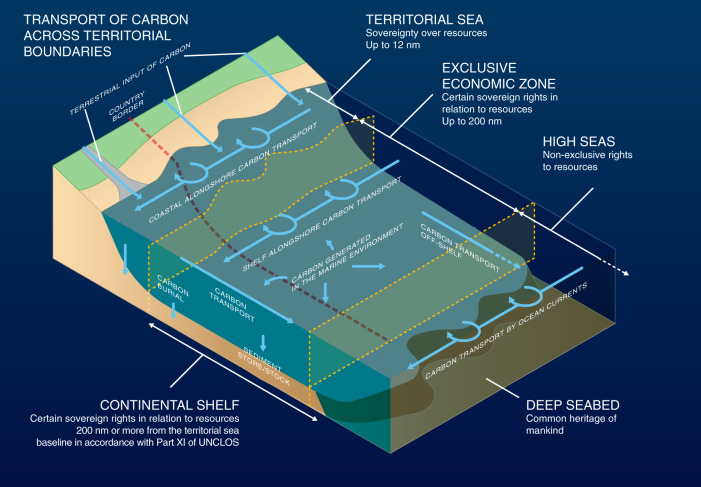
Transport of carbon across territorial boundaries. Input, production, transport and storage pathway of carbon in marine waters, including movement across maritime zones of national jurisdiction: territorial sea, Exclusive Economic Zone (EEZ), continental shelf, high seas, and deep seabed. The rights within each territorial boundary and marine zone relevant to carbon management in the marine environment are summarised for each zone.

This review outlines the uncertainties about carbon sources in the marine environment and carbon transport, and the potential scale of carbon storage in shelf seas, and then examines the potential governance and management of this carbon. Current evidence suggests that, due to transport of carbon in organic matter (i.e. POC) by water currents, the long-term burial of carbon in shelf sea sediments may occur in different territorial waters to those in which it is produced (Fig. 1). This has important consequences for the physical and economic accounting of carbon.

We explore options to address the complexities of organic carbon production, transport and distribution in shelf seas within the technicalities of physical and economic accounting systems for carbon reporting looking at (i) common pool resources^10^ and (ii) the governance and management of carbon sequestered in shelf sea sediments.

We summarise the most relevant insights from biogeochemistry, environmental economics, governance and management of carbon in the marine environment. These elements are all fundamental to understand: (i) the fate of carbon in the marine environment; (ii) the national physical inventory and economic accounting of this carbon; (iii) the economic value of carbon sequestration in the marine environment; and (iv) the national and international governance for the sustainable management of this natural resource. We use the well-studied North Sea^11^ as a real-world example to illustrate the complexities involved. We draw this material together to show the scale of the carbon stores in the marine environment and contemplate how they might be incorporated into carbon management schemes in order to contribute to meeting the 2015 Paris Agreement goals of limiting the global temperature rise well below 2 °C, possibly below 1.5 °C^2^.

## Carbon stocks in shelf sea sediments

The world’s oceans currently take up as much as 25% of anthropogenic carbon dioxide (CO_2_) emissions (1.9 × 10^15^ g y^−1^)^12,13^. While other gases such as nitrous oxide and methane contribute to climate change, we only focus on the fate of CO_2_ in the marine environment because CO_2_ emissions from anthropogenic activities contributed the greatest proportion to the increase of greenhouse gases over the period 1970–2010^14,15^. The CO_2_ is stored throughout the water column where it can be isolated from air–sea exchange for periods of decades to centuries, while other storage occurs via carbon burial in marine sediments.

Most CO_2_ emitted by human activities is trapped within the ocean and stored in the oceanic water column with a residence time of 100–1000s of years^16^. The remaining carbon is stored in marine sediments with residence times of between 100s and millions of years. In ocean sediments on the shelf slope (200–1000 m), or those deeper than >1000 m water depth, storage is extensive^17,18^.

Shelf sediments, defined here as those deposited in <200 m water depth, are less extensive (7.6% of the global marine area)^9^ but globally sequester as much carbon as tropical forests (Table 1). Shelf sediment stores are vulnerable to human activities such as trawling, marine mining and oil and gas exploration, and <200 m water depths have the potential to release CO_2_ into the atmosphere within a year of their disturbance, assuming that the water column is well mixed. The interface between the shelf and land is the location for shallower coastal wetland ecosystems, such as mangrove, tidal marsh and seagrass meadows. Their areal extent is relatively small (<1% of the global area)^19–21^, but they accumulate and store the most carbon per unit area^19^. Because of their locations and characteristics, they are most vulnerable to anthropogenic disturbance and any CO_2_ released can be directly emitted into the atmosphere^20,22^. While there are IPCC guidance for the management of some coastal wetlands ecosystems in national GHG inventories, there is currently a lack of any guidance for regions beyond the coastal zone which would be applicable for management of shelf sea sediments. This is predominantly because of a lack of mapping of the sedimentary environments and the need for further scientific evidence to support the effect of management.

**Fig. 2:**
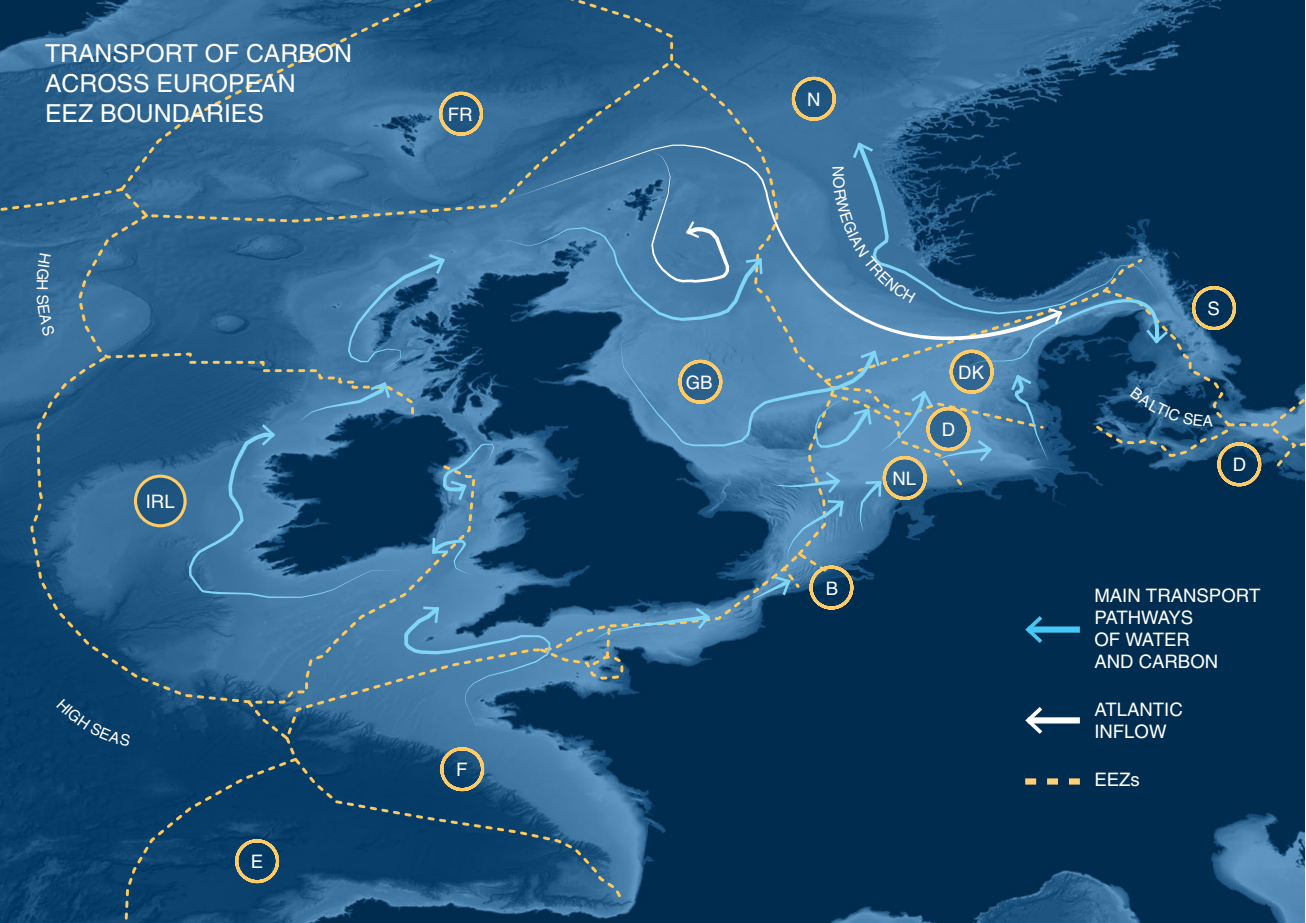
Transport of carbon on the North West European shelf. Illustrating the inflows and transport pathways for carbon in the marine environment, including movement across agreed Exclusive Economic Zone (EEZ) boundaries between [11] countries: (FR) Faroe Islands; (GB) Great Britain; (IRL) Ireland; (F) France; (E) Spain; (B) Belgium; (NL) Netherlands; (D) Germany; (DK) Denmark; (S) Sweden; (N) Norway. Source for coordinates of EEZ boundaries: http://www.marineregions.org/eezmapper.php. The main transport pathways on the North West European shelf^11,26,67^ are based on the main water flow (residual pathways), which will transport particulate and dissolved forms of carbon and sediment. The flows are largely driven by tides, wind driven transport, and density driven flows driven by temperature and salinity differences or by Atlantic inflow (white). They vary in strength, rate and depth depending on season and many cross national EEZ boundaries. EEZ^17^ and bathymetry (blue scale) from http://portal.emodnet-bathymetry.eu/#. Adapted from Hill et al.^26^ and Luisetti et al.^66^.

Every year, the primary productivity of coastal plant communities and phytoplankton in surface waters, as well as the delivery of terrestrial organic matter results in seasonally and annually variable flux of carbon to the sea floor in the form of POC^3^. In cases where there is either restricted transport of carbon inputs by coastal circulation or where continental shelves lie predominantly within the domain of a single country, the carbon can be deposited locally within the jurisdiction of one country. This is the case, for example, on the west coast of the US, the northern Chinese margin seas and Siberian shelf seas. In other cases, depending on the local topography, hydrography and physical characteristics of the water column where the carbon has arrived from or been produced, the carbon can be transported for many hundreds of km and move across national borders where continental shelf areas are shared and along pathways of water circulation and transport. This is the case in the North Sea where UK, Belgian, Dutch, German and Danish borders are all within close proximity of each other, and so water moves rapidly through national waters (Fig. 2). Whether the carbon fractions remain within or move across shared boundaries will also depend on timescales of transport and hydrographic features such as residual currents, stratification and wave events, which dictate surface or near bed transport, or other features such as seasonal jets^23–29^. On shelves, residual currents are typically in the order of 10^−2^ to 10^−1^ m/s, resulting in transport distances of a few km to a few tens of km per day. Particulate transport becomes different and difficult to predict when hydrodynamic conditions are quiet enough, e.g. at neap tides, or during quiet weather, for POC to settle temporarily to the seabed. A proportion may get buried by physical processes or biological activity, and biologically processed until long-term burial or it is remobilised by an erosion event^30–32^. These different scenarios in the transport rates and bed interaction of POC illustrate that transit times across boundaries can be days to years depending on hydrography (including seasonal and inter annual variability), sedimentology, biogeochemical processes and proximity to boundaries. From a carbon storage and Exclusive Economic Zone (EEZ) point of view, an approach could be to, for a particular case, identify different zones where, in comparison to burial rate, transport of mobile POC (in the water column or as bed-load) is likely to be quick, intermittent, slow or very slow to negligible. Such areas would have different status in terms of the transport of POC generated in situ (or moved in from elsewhere) and hence associated sensitivity to anthropogenic disturbance in the sense of carbon storage.

We focus on the North Sea example to show how challenging accounting for governance and management of carbon stocks in the marine environment can be. However, worldwide, most other shelf seas or continental shelf margins that are bordered by multiple countries present similar challenges.

The fate of carbon is driven by hydrodynamics and sedimentary processes. Throughout their vertical and horizontal transport, different carbon fractions can be remineralised, depending on their resistance to decomposition, the hydrodynamics and the biological communities present. The proportion of each of these sources that is delivered to the sediment, as well as the rate at which they accumulate and are stored, can vary with environmental conditions. In the North Sea, one of the main well-established current patterns carries water and POC anti-clockwise (Fig. 2), from the north-west along the UK and northern European coast to the main location of burial in the deep Norwegian trench^11,26,33^. Thus, depending on the regional geomorphology and hydrography, carbon removed from the atmosphere in one location (e.g. one specific country territorial sea or EEZ), may be processed during transport, causing the release of CO_2_, sedimentation of carbon or advection, with the remaining carbon being ultimately stored in a different location (e.g. another specific country EEZ). All shelf sea systems are important stores of organic carbon, but the sites of storage within these systems varies widely with their different geographic settings^18,34,35^. Storage of riverine or coastal vegetative material is influenced by shelf width and circulation^36^. The North Sea is an example of a geological passive margin with a wide shelf. Convergent plate margins (for example Taiwan), by contrast, create narrow shelves where fluvial or coastally derived carbon may be exported off shelf and stored at depths >200 m^37^.

When organic matter reaches the sea floor as POC, whether produced locally or advected there, it contributes to carbon that has already accumulated there. Some of the organic matter will be fresh and/or easily decomposed, while some will be refractory or older and more resistant to decomposition^31,38^. Various physical and biological mechanisms lead to the incorporation and eventual burial of the deposited organic carbon^30,31,39–41^. Thus, the amount of carbon stored in marine sediments for significant periods of time depends on the detrital carbon concentration in the water, accumulation rate in the sediment, controls on remineralisation rates and bio-physical pathways within the bed^17,42^. Direct or indirect disturbances of the seabed or changes in water column conditions can affect many of these processes and thus the overall carbon stock, storage rates and capacity^17,30,43–45^. Anthropogenic activities in the territorial waters of a country containing significant carbon stocks could result in the release of carbon that has been undisturbed for centuries or longer. Such carbon could subsequently be re-deposited locally, transported by currents and deposited elsewhere, or be remineralised, potentially leading to CO_2_ release into the atmosphere. The likelihood of this release occurring depends on the environmental conditions that generated that stock. The fate of this carbon (i.e. whether it is retained within a nation’s EEZ) will ultimately be controlled by biologically and physically mediated transport processes. Thus, features like fjords may enhance local deposition, submarine canyons may enhance off shelf transport^43,46^ and strong current flows may enhance long range transport.

## Existing accounting frameworks

One of the IPCC’s actions is to support the UNFCCC by providing standardised and internationally agreed methodologies for national GHG inventories. In the 2006 IPCC Guidelines^47^, the agriculture, forestry and other land use (AFOLU) sector is the only Inventory sector in which it is possible for countries to report both GHG emissions and sinks. When the 2006 IPCC Guidelines were developed, coastal wetlands were not included, but the 2013 Supplement to the 2006 IPCC Guidelines for National Greenhouse Gas Inventories: Wetlands (Wetlands Supplement)^48^ addressed this omission and acknowledged the role of mangroves, tidal marshes and seagrass meadows in helping mitigate climate change through a nature based solution^49,50^. However, in any national GHG inventory, their country’s reporting boundary is defined separately and may not currently extend below the tidal high-water level. Thus, their reporting boundary excludes shelf seas, and may have to be extended seawards to include the maximum depth of seagrass vegetation^51^. Although the latest IPCC assessment^16^ mentions the relevance of the carbon stored in coastal and marine vegetated ecosystems, the shelf sea and the deep ocean, at present accounting guidelines do not include most of these ecosystems. We illustrate the challenges of producing guidelines for marine ecosystems by briefly outlining relevant aspects of the current guidelines covering coastal wetlands.

The storage and loss of atmospheric CO_2_ from coastal wetlands has multiple causes. In the IPCC guidelines they are captured by a gain or loss of above and below ground biomass, deadwood, litter and soil organic carbon in mineral and organic soils, regardless of whether these changes occur on-site or offsite. While changes in areal extent of land-use are identified in GHG inventories, only emissions and removals connected with managed lands are reported. Naturally occurring emissions and removals are not included.

In most Inventory sectors, the distinction between anthropogenic and naturally occurring emissions is clear, but for AFOLU (including coastal ecosystems) it is more difficult to discern whether a specific management activity is the unique driver of any GHG emission or removal. Given this challenge, the IPCC developed a managed land proxy, which identifies those land areas where anthropogenic emissions dominate. Countries can report in their national inventories all GHG fluxes from land defined as managed land, including coastal wetlands^52^. It is accepted that this managed land proxy may only approximate what occurs in reality, as it is assumed that the natural background of biologically driven fluxes averages out over time and space and does not separate direct from indirect anthropogenic effects, such as those attributed to climate change.

The concept of the managed land proxy has been interpreted in different ways by countries when compiling their national inventories. In fact, most countries do not report how they apply the managed land proxy, and those that do, simply assign all stated land-uses to managed land, regardless of whether there is evidence that anthropogenic emissions dominate^53^. It has been acknowledged that practical methodologies must be developed that separate direct from indirect human-induced and natural effects^54,55^, but this has not yet been achieved^56^. Until such methodologies are established, emissions and removals on managed land are assumed to be only due to direct human management activities. In coastal wetlands, carbon emissions and removals^57,58^ may be driven by activities that occur within the wetland itself or be driven by changes in adjacent land sectors, such as upstream changes in land management. In some instances, i.e. the Mississippi river network^52^, the location of cause and effect were both within the national reporting boundary. This may not always be the case and in regions bounded by a number of different countries, management activities within the national reporting boundary of one country may result in emissions or removals within the national reporting boundary of a different country.

Resulting from national reporting for the UNFCCC Inventories, the IPCC represents the most extensive source of carbon flow data for economic accounting purposes. Carbon flow data captured within GHG inventories provide the necessary evidence to complement the current System of National Accounts (SNA) (i.e. a set of tables using exchange-market values as unit of measure). SNA tables provide a measure of the economic activities in a country to determine, among other indicators, their gross domestic product (GDP), and may be complemented by carbon stock physical accounts (i.e. a set of tables using natural science unit of measures)^59^ to measure the economic contribution of carbon management strategies.

The System of Environmental-Economic Accounting (SEEA) is an economic accounting framework aimed at integrating environmental data with measures of economic activities. The SEEA Central Framework (CF)^60^ establishes the rules to account for the dependency of economic activities (e.g. agriculture, mineral extraction, energy production) on natural capital assets or stocks (e.g. land, oceans, soil). The SEEA Experimental Ecosystem Accounting (EEA)^61^ sets the framework to link ecosystem services flows (e.g. water flows, crops) to economic sectors/activities organized in a set of tables and accounts. For appropriate reporting, CF and EEA require data on: (i) physical terms, through spatially explicit ecosystem maps and accounting tables (ii) economic measures, where the physical data are monetized with values (e.g. exchange prices).

The CF and EEA represent an important step in officially recognizing ecosystem services benefits, such as carbon sequestration, to society. EEA^61^ acknowledges carbon as one of the ecosystem assets requiring more research, and in the Technical Recommendations it is defined as a thematic account^59^; however, the carbon stored in the marine environment is excluded. In the EEA, carbon sequestration is defined as the process encompassing both the service of sequestration and storage of carbon, highlighting that “both services are important for ecosystem management and therefore for ecosystem accounting”^61^. However, Edens et al. explicitly subdivide carbon sequestration into two components: carbon storage (carbon burial) defined as an asset stock (i.e. the carbon store/sink); and carbon sequestration (carbon capture), defined as the marginal change to the stock due to the annual accumulation^62^. Therefore, this definition of carbon sequestration differs from that in the natural sciences, and this ambiguity can lead to misunderstanding and misreporting of the economic value of carbon in economic accounts. For example, in the latest SEEA documents, carbon sequestration is considered a final service, whereas in the latest Common International Classification of Ecosystem Services (CICES)^63^ it is considered as an intermediate service. Thus, although measurement and valuation of both flow and stock measures can contribute to a more cost-effective climate policy, for a comprehensive measurement of changes in wellbeing over time, the definition of the carbon sequestration process that includes both capture and storage of carbon, providing climate regulation as a final service^64^ and the global benefit of a healthy climate, should be adopted.

## Carbon economics and governance in the marine environment

The economic value of the ecosystem service of carbon sequestration has been estimated in several parts of the world for saltmarshes, mangroves and seagrass meadows^65^. Carbon in shelf sea sediments, however, has yet to receive the same attention, although there are some emerging studies that explore this possibility^8^. Anthropogenic disturbance of shelf sea sediments may exacerbate climate change effects and reduce human wellbeing due to potential future welfare damages estimated in the range of billions of US dollars^66^. The estimation of the cost of carbon to society is best expressed as a range of economic values, although the basis for its estimation is a matter of debate between exchange price and cost-based positions. It is worth noting that there is growing mining pressure on seabed resources within the 200-mile national economic zone boundaries^67^. So far, the main concern has been focused on the international waters, such the High Seas, which are outside and beyond national jurisdiction and cover about 60% of the ocean^68^, and where interactions with the seabed and water column are subject to a range of bespoke governance frameworks, underpinned by non-exclusive rights enjoyed by all countries. However, currently, under the International Seabed Authority (ISA)^69^ voluntary commitments for the sustainable management of mining in the High Seas are starting to emerge.

Coastal and marine vegetated ecosystems are geographically defined. Therefore, their jurisdictional status within the territorial boundaries of a country and the ownership within precise national reporting boundaries are clear, facilitating the accountably of their management. Financial resources for the conservation of these ecosystems may come both from the public and the private sector. For example, national and international regulated and voluntary markets for carbon credits trading now exist around the globe^65^, including the European Union Emissions Trading System (EUETS). However, since the carbon sequestered in organic matter (i.e. POC) may be transported in the water column crossing several territorial waters and jurisdictions, POC could be considered a mobile common pool resource^10^.

There are many existing examples of mobile and transboundary resource (or pollutant) governance frameworks, which may provide a reference point for an innovative one aimed to manage carbon sequestration in the marine environment, through POC, and related carbon stocks. However, uncertainties around the underpinning science, lack of new readily available technologies for monitoring, and political difficulties arising from implementation and action at the local level of international agreements seem to limit effective governance of common pool resources and global benefits. For example, the International Commission for the Conservation of the Atlantic Tuna (ICCAT) has been considered an institutional failure primarily because of the monitoring challenges for implementing an appropriate command and control policy^70^. An assessment of the National Biodiversity Strategies And Action Plans (NBSAPs) towards the achievement of the Aichi Biodiversity Targets of the Strategic Plan for Biodiversity 2011–2020 within the Convention of Biological Diversity^71^ found that national biodiversity targets are lower than the more ambitious Aichi Biodiversity Targets^72^. Lack of political coordination at the local level together with a need to reform on existing legal frameworks seem to have limited the success of the Strategic Plan^73^. In the marine environment, the new United Nations Convention on the Law of the Sea (UNCLOS) implementing agreement on ‘the conservation and sustainable use of marine biological diversity of areas beyond national jurisdiction’^74^ gives hope. In the 1980’s, to deal with the Baltic Sea eutrophication due to a high concentration of nutrients discharged into the sea through the rivers of several countries, the Baltic Marine Environment Protection Commission–Helsinki Commission (HELCOM) was formed. However, HELCOM measures have not led to significant improvements due to the isolation and long response time of the Baltic system. Also, the co-existence of national, European and international governance within the same geographical area limits the effective implementation of regional environmental governance in the Baltic Sea^75^.

## Main challenges for assessment and management of carbon

Large uncertainties regarding the sources and age (e.g. fossil terrestrial (geologic) or recently created terrestrial or marine carbon)^34,37,76^, amounts and timescales of carbon storage, the carbon transport pathways, and the impact of the different human activities on net carbon storage still remain^77^. Some anthropogenic activities can even have counter-acting effects on carbon stocks and storage. Interacting drivers make the net effects as yet difficult to predict both in space and time. Increased water stratification can isolate bottom waters and cause deoxygenation, often called dead zones, which changes the carbon breakdown pathways to slower processes, potentially increasing storage. Eutrophication and the enhancement of primary production can also cause formation of these dead zones^78^. However, this might not necessarily be considered beneficial, as the negative impacts of eutrophication on the biota (for example the lack of oxygen) and ecosystem (changes in food webs) may outweigh the carbon storage benefits. Other factors such as temperature increase, productivity decrease, input changes and ocean acidification will also influence carbon storage. In terms of management, when making carbon stock assessments and calculating burial fluxes, the depth to which activities disturb the sediment defines the depth of sediment where data should be acquired. For example, 10 cm is a depth to which disturbance by human activities, such as trawling, is likely and is a depth for which most carbon measurements exist^79^. However, there are stores of often much older carbon deeper in the sediments, depths within the sediments which make them less likely to be disturbed^17^.

Lack of robust biophysical data for the assessment of carbon sequestration fluxes and carbon stocks will translate in uncertain economic valuations. Uncertainty regarding the origin of the carbon fraction in the marine environment may risk double counting the benefits of the service of carbon sequestration. Monitoring of the carbon stock is fundamental to verify any occurring anthropogenic disturbance of shelf sea sediments and associated risk of releasing previously stored carbon. However, a country interested in accounting for the carbon stock in their territorial waters might need to budget substantial costs for monitoring the extent of the carbon stock and its fluxes, which ultimately provide common benefits. Regional agreements, like OSPAR, that build towards a standardised set of indicators and shared data collection for national natural capital accounting can help in this context^80^.

Although some countries, including the UK, are contemplating recording the economic value of shelf sea sediments in their marine natural capital accounts^81^, uncertainty around the origins of POC and the vulnerability of carbon stocks limit the accuracy of the economic valuation. The EEA revision document^62^ considers carbon accounting still problematic, and for accounting of marine stocks and flows it suggests looking at how carbon accounting is done on land. However, translation of lessons learnt from land accounting to ocean accounting is not straightforward. Current UNFCCC reporting boundaries for the AFOLU sector distinguish between managed and unmanaged land^47^, but this definition is not aligned with the distinction between cultivated and non-cultivated land in the SNA and CF.

IPCC guidance on GHG emissions and removals is relatively well established in coastal wetland settings under national jurisdiction, but for the shelf sea sediments new guidelines would need to be devised. First, inclusion of most of the shelf seas will require a re-definition of a country’s reporting boundary and to ensure time series consistency, any changes must also be applied to previous years’ inventories; second, within these new reporting boundaries, the extent of managed and unmanaged lands would need to be mapped or an activity-based approach taken to assessing emissions and removals. Third, further data and methodological guidance would be required for assessing GHG emissions and removals associated with management activities affecting the carbon stores, including carbon stock as well as flow accounts^82^. Fourth, guidance would be required for emissions and removals associated with a particular activity to decide whether to include them in the country where the activity originated (e.g. sediment disturbance) or in the country where the disturbed sediment is moved to and eventually accumulates. In this new setting the sediment may be stored or may again become vulnerable to further change. This last challenge is particularly relevant to a marine setting where the flows of carbon can move across national reporting boundaries.

Central would be the provision of data to support such methodological development for specific management activities in shelf seas. Also, the provision of further guidance would be needed on whether emissions and removals associated with a particular activity should be included in the sediment inventories of the country where the activity occurred, or in the inventory of another country where some proportion of the disturbed sediment eventually resided and where it may be finally buried, and yet still vulnerable to further management activities. Activities which do not respect national reporting boundaries are already being identified, such as the change in sediment delivery in the coastal wetlands of one country caused by upstream dam construction in a different country^52^. In shelf seas, similar situations may emerge and could be an important component of a country’s inventory^8^. Indirect anthropogenic effects on carbon located in coastal and shelf seas, including changing inputs from land, ocean productivity, greater ocean stratification driven by surface warming, ocean acidification, changes to ocean circulation and sea level rise^16^, are potentially significant, and yet any policy framework for areas beyond national reporting boundaries has yet to emerge.

Economic accounting of carbon in marine settings is particularly challenging because the definition of marine assets is in development and the related biophysical information is limited. The EEA revision process^63^ explicitly acknowledges the need to expand the EEA to the marine environment through Ocean Accounts. The UN Statistical Commission has now endorsed an ongoing process led by the UN Economic and Social Commission for Asia and the Pacific (ESCAP) and the Global Ocean Accounts Partnership to develop specific technical guidance for ocean accounting consistent with the general principles and approaches of the SEEA^83^. Marine data are not always readily available in central databases, and it is therefore sometimes difficult to extract required information. The use of international data integration and sharing would help to overcome these limitations. A complementary account network providing information coming from other sources outside standard accounts, such as the IPCC inventories, may also be considered^84,85^. Other challenges in harmonising Ocean Accounts with the EEA arise from the mobile and transboundary nature of the ocean processes. One option may be to develop multi-regionally accounting via a multi-regional input–output analysis that overcomes the limit of territorial boundaries and records the transnational economic and environmental activities^86^. However, the existing global multi-regional input–output datasets^87^ do not currently include ocean and marine ecosystems. For strict physical accounting purposes, the transboundary issue relates mainly to the difficulty of defining spatial units and ecosystem types. In the marine environment, an option could be to develop a global spatial data infrastructure with common spatial grid referencing and ecosystem typology hierarchy to aggregate spatial units. This development would require a common international definition and classification of marine ecosystems and related services. Also, economic accounts require that different economic sectors impacting on, or benefitting from, an ecosystem service are identified and monitored. However, with reference to carbon, beneficiaries (of the carbon sequestration service) and producers (of CO_2_ emissions) are both locally and internationally located.

Ocean accounting practices, inclusive of shelf sea sediments, have therefore the potential to provide continuous ecosystem monitoring and a standardised set of ecosystem condition indicators, possibly georeferenced at different scales for national planning^88^. Depending on how these accounting data are collected and analysed, they could contribute to improved understanding of ecosystem use and greater transparency on ecosystem use and management^89^. This may also enable improved understanding and measurement of the often ambiguous synergies and trade-offs between climate change impacts, climate change mitigation and SDG targets^90^, including the positive and negative interrelationship between the targets of SDG 14 (life below water) and SDG 13 (climate action)^91^.

The location and definition of who owns and is responsible for any given carbon stock in the marine environment is essential for safeguarding it. This becomes even more pressing when considering that globally the positions of around half of all maritime boundaries are still contested, or that there are large areas without sovereignty, such as the High Seas. If the carbon initially accumulating in the territorial waters of several different countries is disturbed and is redeposited outside their jurisdiction, the management and protection of the carbon stock may be transferred to the responsibility of the country in which it is ultimately stored. In this scenario, and in a scenario like the High Seas, where non-exclusive rights are enjoyed by all countries, a range of so-called free-riding behaviours might arise. Free riding over shelf seas may result in a tragedy of the commons^92^ because the disturbance of a carbon stock by anthropogenic activities for the benefit of some will be to the detriment of the global mitigation benefits of carbon sequestration. Scenarios of inadequate management of natural resources can be a consequence of unclear delineation of natural resource rights and responsibilities, and non-existing or inadequate existing governance and related accountability.

Ambiguous natural resource ownership and the challenging monitoring of POC and related carbon stock accumulated in shelf sea sediments are possibly the reasons why these carbon stores have not yet been considered within carbon credit trading schemes and for nationally determined contributions (NDCs)^8^. Under the 2015 Paris Agreement, nation states are required to submit voluntary emission reduction pledges in the form of NDCs^93^ which are revised every 5 years; each revision requires an improved mitigation contribution^94^. NDCs vary greatly in format, scope and structure^95^. A 2017 review indicated some NDCs refer to carbon sequestration in marine ecosystems, for example in mangroves and carbon fractions from terrestrial and marine sources, but none to carbon stocks in shelf seas^96^.

In the North Sea, at the regional level, some international agreements already exist for the protection of marine areas. The UNEP Regional Seas Programme proposes a shared areas approach^97^. This arrangement might reduce the local scale issue of ecosystem management to reach global benefits. So far, the UNEP Programme has 18 Regional Seas Programmes based on Action Plans each underpinned by a Regional Sea Convention (legally binding). Within each regional convention there is scope for protocols which can address specific issues, one of which could be the mobile nature of POC and the ecosystem service of carbon sequestration it provides. Regional Seas Programmes could also ensure the conservation of the carbon stocks, for example addressing compensation measures for the restriction of activities which may disrupt the stock accumulation process. The UNEP administer some but not all the Regional Programmes; the North-East Atlantic Region is coordinated by OSPAR.

## Towards an integrated accounting and governance framework

Similar to the case for migratory fish, we have investigated the fate of transboundary carbon from science to policy in its itinerant ecosystem processes from carbon capture in the water column to the final accumulation of the particulate portion in shelf sea sediments to consider: (1) how ownership of carbon stocks in shelf sea sediments is distributed; (2) how these carbon stocks should be measured and registered in national carbon accounts; and (3) what arrangements can be made to ensure the conservation of the carbon stocks.

Our review has highlighted the need to identify supranational mechanisms capable of governing the challenges presented, especially the challenge on limited data availability and standardisation. We highlight the need to develop new methods for ocean physical and monetary accounting and incorporate this new ocean accounting guidance, which includes the accounting of carbon in shelf sea sediments, into new regulatory frameworks and international agreements.

Meeting these will help address preservation of carbon stores in the marine environment and foster collaboration between neighbouring countries responsible for the interconnected and collective impacts of economic activities on the global benefit of a healthy climate. This may lead the way towards an accurately defined share of carbon stock accounting between all countries that have a role documenting and assisting with the management of shelf sea storage of carbon that crosses the territorial boundaries of the marine waters it was captured in. This would also require that the mitigation value of the carbon stocks in marine settings was apportioned in some proportional manner to those nations whose activities have generated some of the carbon now stored in a carbon store located in a different jurisdiction.

## Supplementary information

Peer Review File

## References

[1] Virto LR, Weber J-L, Jeantil M (2018). Natural capital accounts and public policy decisions: findings from a survey. Ecol. Ec..

[2] United Nations. Framework convention on climate change adoption of the Paris Agreement. *21st Conference of the Parties* (United Nations, Paris, 2015).

[3] Liénart C (2018). Dynamics of particulate organic matter composition in coastal systems: forcing of spatio-temporal variability at multi-systems scale. Prog. Oceanogr..

[4] Pedrosa-Pàmies R (2015). Composition and sources of sedimentary organic matter in the deep eastern Mediterranean Sea. Biogeosciences.

[5] Van der Voort TS (2018). Deconvolving the fate of carbon in coastal sediments. Geophys. Res. Lett..

[6] da Silva Copertino, M. Add coastal vegetation to the climate critical list. *Nature* **473**, 255 (2011). 10.1038/473255a 21593818

[7] Cooley, S. R. et al. Overlooked ocean strategies to address climate change, *Global Environ. Change* **59**, 101968 (2019).

[8] Avelar, S., van der Voort, T. S. & Eglinton, T. I. Relevance of carbon stocks of marine sediments for national greenhouse gas inventories of maritime nations. *Carbon Balance Manag.* **12**, 10 (2017). 10.1186/s13021-017-0077-x PMC5423874 28474331

[9] Holt J (2017). Prospects for improving the representation of coastal and shelf seas in global ocean models. Geosci. Model Dev..

[10] Ostrom, E., Gardner, R. & Walker, J. (eds). *Rules, Games, and Common-Pool Resources* (Univ. of Michigan Press, Ann Arbor, 1994).

[11] Legge, O. et al. Carbon on the Northwest European Shelf: contemporary budget and future influences. *Front. Mar. Sci.* **7**, 143 (2020). ***This paper presents a new budget synthesis of carbon cycling in the North Sea***.

[12] IPCC. *Contribution of Working Group I to the Fourth Assessment Report of the Intergovernmental Panel on Climate Change* (eds Solomon, S. et al.) (Cambridge University Press, Cambridge, UK and New York, NY, USA, 2007).

[13] Le Quéré, C. et al. Global carbon budget 2018. *Earth Syst. Sci. Data*10.5194/essd-10-2141-2018 (2018).

[14] IPCC Summary for Policymakers. In *Climate Change 2013: The Physical Science Basis. Contribution of Working Group I to the Fifth Assessment Report of the Intergovernmental Panel on Climate Change* (eds Stocker, T. F. et al.) (Cambridge University Press, Cambridge and New York, 2013).

[15] IPCC Summary for Policymakers. In *Climate Change 2014: Mitigation of Climate Change. Contribution of Working Group III to the Fifth Assessment Report of the Intergovernmental Panel on Climate Change* (eds Edenhofer, O. R. et al.) (Cambridge University Press, Cambridge and New York, 2014).

[16] IPCC. *Special Report on the Ocean and Cryosphere in a Changing Climate* (eds Pörtner, H.-O. et al.) (2019).

[17] Burdige, D. J. The preservation of organic matter in marine sediments: controls, mechanisms and an imbalance in sediment organic carbon budgets? *Chem. Rev.* **107**, 467–485 (2007). ***This is a major review of sediment organic carbon processing***. 10.1021/cr050347q 17249736

[18] Atwood, T. B., Witt, A., Mayorga, J., Hammill, E. & Sala, E. Global patterns in marine sediment carbon stocks. *Front. Mar. Sci.* **7**, 1 (2020).

[19] Taillardat, P., Friess, D. & Lupascu, M. Mangrove blue carbon strategies for climate change mitigation are most effective at the national scale. *Biol. Lett.* **14**, 20180251 (2018). 10.1098/rsbl.2018.0251 PMC6227866 30355678

[20] Duarte CM, Dennison WC, Orth RJ, Carruthers TJ (2008). The charisma of coastal ecosystems: addressing the imbalance. Estuaries Coasts.

[21] Duarte C (2013). The role of coastal plant communities for climate change mitigation and adaptation. Nat. Clim. Change.

[22] Pendleton, L. et al. Estimating global “blue carbon” emissions from conversion and degradation of vegetated coastal ecosystems. *PLoS ONE* **7**, e43542 (2015). 10.1371/journal.pone.0043542 PMC3433453 22962585

[23] Otto L (1990). Review of the physical oceanography of the North Sea. Neth. J. Sea Res..

[24] Simpson, J. H. & Sharples, J. *Introduction to the Physical and Biological Oceanography of Shelf Seas*, 413pp (Cambridge University Press, 2012).

[25] Soulsby, R. *Dynamics of Marine Sands: A Manual for Practical Applications* 249pp (Thomas Telford, 1997).

[26] Hill, A. E. et al. Thermohaline circulation of shallow tidal seas. *Geophys. Res. Lett.* **35**, 10.1029/2008GL033459 (2008).

[27] Brown J (2003). Observation of the physical structure and seasonal jet-like circulation of the Celtic Sea and St. George’s channel of the Irish. Cont. Shelf Res..

[28] Fernand, L. et al. The Irish coastal current: a seasonal jet-like circulation, *Cont. Shelf Res*. **26**, 1775–1793 (2006).

[29] Bristow LA (2013). Tracing estuarine organic matter sources into the southern North Sea using C and N isotopic signatures. Biogeochemistry.

[30] Huettel M, Berg P, Kostka JE (2014). Benthic exchange and biogeochemical cycling in permeable sediments. Annu. Rev. Mar. Sci..

[31] Middelburg JJ (2018). Reviews and syntheses: to the bottom of carbon processing at the seafloor. Biogeosciences.

[32] Couceiro F (2013). Impact of resuspension of cohesive sediments at the Oyster Grounds (North Sea) on nutrient exchange across the sediment–water interface. Biogeochemistry.

[33] Wilson RJ, Speirs DC, Sabatino A, Heath MR (2018). A synthetic map of the north-west European Shelf sedimentary environment for applications in marine science. Earth Syst. Sci. Data.

[34] Bianchi TS, Blair N, Burdige D, Eglinton TI, Galy V (2018). Centers of organic carbon burial at the land-ocean interface. Org. Geochem..

[35] Bauer J (2013). The changing carbon cycle of the coastal ocean. Nature.

[36] Sharples, J., Middelburg, J. J., Fennel, K. & Jickells, T. D. What proportion of riverine nutrients reaches the open ocean? *Global Biogeochem. Cycles*, **31**, 39–58 (2017).

[37] Kao SJ (2014). Preservation of terrestrial organic carbon in marine sediments offshore Taiwan: mountain building and atmospheric carbon dioxide sequestration. Earth Surf. Dyn..

[38] Burdige, D. J. *Geochemistry of Marine Sediments* 1–609 (Princeton University Press, 2006).

[39] Aller RC (1994). Bioturbation and remineralization of sedimentary organic matter: effects of redox oscillation. Chem. Geol..

[40] Aller, R. C. In *The Benthic Boundary Layer: Transport Processes and Biogeochemistry* (eds Boudreau, B. & Jørgensen, B. B.) 269–301 (Oxford Press, 2001).

[41] Teal LR, Bulling MT, Parker ER, Solan M (2008). Global patterns of bioturbation intensity and mixed depth of marine soft sediments. Aquat. Biol..

[42] Arndt S (2013). Quantifying the degradation of organic matter in marine sediments: a review and synthesis. Earth-Sci. Rev..

[43] Levin LA, Sibuet M (2012). Understanding continental margin biodiversity: a new imperative. Annu. Rev. Mar. Sci..

[44] Pusceddu A (2014). Chronic and intensive bottom trawling impairs deep-sea biodiversity and ecosystem functioning. PNAS.

[45] Paradis S (2019). Organic matter contents and degradation in a highly trawled area during fresh particle inputs (Gulf of Castellammare, southwestern Mediterranean). Biogeosciences.

[46] Maier, K. L. et al. Sediment and organic carbon transport and deposition driven by internal tides along Monterey Canyon, offshore California. *Deep Sea Res. Part I: Oceanogr. Res. Pap.* **153**, 103–108 (2019).

[47] IPCC. *IPCC Guidelines for National Greenhouse Gas Inventories—A Primer*. Prepared by the National Greenhouse Gas Inventories Programme (eds Eggleston, H. S., Miwa, K., Srivastava, N. & Tanabe, K.) (IGES, Japan, 2008).

[48] IPCC. *2013 Supplement to the 2006 IPCC Guidelines for National Greenhouse Gas Inventories: Wetlands* (eds Hiraishi, T. et al.) (IPCC, Switzerland, 2014).

[49] Schuerch M (2018). Future response of global coastal wetlands to sea-level rise. Nature.

[50] Griscom BW (2017). Natural climate solutions. PNAS.

[51] Troxler, T., Kennedy, H., Crooks, S. & Sutton-Grier, A. In *A Blue Carbon Primer: The State of Coastal Wetlands Carbon Science, Practice and Policy* (eds Windham-Myers, L., Crooks, S. & Troxler, T.) (CRC Press, Boca Raton, Fl, 2018).

[52] Crooks S (2018). Coastal wetland management as a contribution to the US National Greenhouse Gas Inventory. Nat. Clim. Change.

[53] Ogle, S. M. et al. Delineating managed land for reporting national greenhouse gas emissions and removals to the United Nations framework convention on climate change. *Carbon Bal. Manag.* **13**, 9 (2018). 10.1186/s13021-018-0095-3 PMC5974992 29845384

[54] IPCC. *Current Scientific Understanding Of The Processes Affecting Terrestrial Carbon Stocks And Human Influences Upon Them*. IPCC Meeting on Expert Meeting Report (eds David Schimel, D. & Martin Manning, M.), Geneva, Switzerland, 21–23 July (2003).

[55] Canadell JG (2007). Contributions to accelerating atmospheric CO_2_ growth from economic activity, carbon intensity, and efficiency of natural sinks. Proc. Natl Acad. Sci. USA.

[56] Krug, J. H. A. Accounting of GHG emissions and removals from forest management: a long road from Kyoto to Paris. *Carbon Bal. Manag.* **13**, 1 (2018). 10.1186/s13021-017-0089-6 PMC5768587 29330699

[57] Lovelock CE (2017). Assessing the risk of carbon dioxide emissions from blue carbon ecosystems. Front. Ecol. Environ..

[58] Thorhaug A, Poulos HM, Lopez-Portillo J, Ku TCW, Berlyn GP (2017). Seagrass blue carbon dynamics in the Gulf of Mexico: stock, losses from anthropogenic disturbance, gains through seagrass restoration. Sci. Total Environ..

[59] United Nations. *Technical Recommendations in Support of the System of Environmental – Economic Accounting 2012 – Experimental Ecosystem Accounting*, 193 (White Cover Publication, United Nations, 2017).

[60] United Nations, European Commission, Food and Agriculture Organization, International Monetary Fund, Organisation for Economic Co-operation and Development, and the World Bank. *System of Environmental-Economic Accounting 2012. Central Framework* (2014).

[61] United Nations, European Commission, Food and Agriculture Organization, Organisation for Economic Co-operation and Development, and the World Bank. *System of Environmental-Economic Accounting 2012: Experimental Ecosystem Accounting*—final, official publication (2014).

[62] Edens, B., Elsasser, P. & Ivanov, E. Discussion paper 6: Defining and valuing carbon related services in the SEEA EEA. Paper submitted to the Expert Meeting on Advancing the Measurement of Ecosystem Services for Ecosystem Accounting, New York, 22–24 January 2019 and subsequently revised. https://seea.un.org/events/expert-meeting-advancing-measurement-ecosystem-services-ecosystem-accounting (2019).

[63] European Environment Agency. *Natural Capital Accounting in Support of Policymaking in Europe—A Review Based on EEA Ecosystem Accounting Work*. EEA Report No 26/2018 (Publication Office of the European Union, Luxembourg, 2019). ***Natural capital accounting provides evidence on ecosystem trends in a structured and integrated manner that allows for analysis of environment-economy interactions, with diverse entry points into policymaking processes***.

[64] UK National Ecosystem Assessment. *The UK National Ecosystem Assessment: Follow-on (UK NEA-FO)* (UNEP-WCMC, LWEC, UK, 2014). ***This report applies the ecosystem services approach and balance sheet decision support system to environmental policy***.

[65] Thomas S (2014). Blue carbon: knowledge gaps, critical issues, and novel approaches. Ecol. Econ..

[66] Luisetti T (2019). Quantifying and valuing carbon flows and stores in coastal and shelf ecosystems in the UK. Ecosyst. Serv..

[67] Santos MM (2018). The last frontier: coupling technological developments with scientific challenges to improve hazard assessment of deep-sea mining. Sci. Total Environ..

[68] Orbach, M. Beyond the freedom of the seas: ocean policy for the third millennium. *Oceanography* **16**, 20 (2003).

[69] International Seabed Authority (ISA) Voluntary Commitments to Support Implementation of SDG14: https://www.isa.org.jm/isa-voluntary-commitments.

[70] Epstein, G., Nenadovic, M. & Boustany, A. Into the deep blue sea: commons theory and international governance of Atlantic Bluefin Tuna. *Int. J. Commons* **8**, 277–303 (2014).

[71] United Nations. *Convention of Biological Diversity*, https://www.cbd.int/sp/ (1992). 10.1016/s0378-8741(96)90036-7 9213623

[72] CBD – Subsidiary Body on Implementation. Second meeting Montreal, Canada, 9–13 July 2018. Item 3 of the provisional agenda. *Analysis of the Contribution of Targets Established by Parties and Progress Towards the Aichi Biodiversity Targets: Note by the Executive Secretary* (Montreal, Canada, 2018).

[73] UNEP. *Assessment of post-2010 National Biodiversity Strategies and Action Plans* (Nairobi, Kenya, 2018).

[74] UN General Assembly Intergovernmental conference on an international legally binding instrument under the United Nations Convention on the Law of the Sea on the conservation and sustainable use of marine biological diversity of areas beyond national jurisdiction. Fourth session New York, 23 March–3 April 2020 - Revised draft text of an agreement under the United Nations Convention on the Law of the Sea on the conservation and sustainable use of marine biological diversity of areas beyond national jurisdiction. *Note by the President* (2020).

[75] Scharin H (2016). Processes for the sustainable stewardship of marine environments. Ecol. Econ..

[76] Hilton RG (2008). Tropical-cyclone-driven erosion of the terrestrial biosphere from mountains. Nat. Geosci..

[77] Wakelin, S. L. et al. Modeling the carbon fluxes of the northwest European continental shelf: validation and budgets. *J. Geophys. Res.* **117**, 10.1029/2011JC007402 (2012). ***This study provides the most complete model-derived carbon fluxes for the northwest European continental shelf to date***.

[78] Keil R (2017). Anthropogenic forcing of carbonate and organic carbon preservation in marine sediments. Annu. Rev. Mar. Sci..

[79] Diesing M (2017). Predicting the standing stock of organic carbon in surface sediments of the North–West European continental shelf. Biogeochemistry.

[80] OSPAR Assessment Portal. *Socioeconomics of the OSPAR Maritime Area—Towards and Assessment Framework*. https://oap.ospar.org/en/ospar-assessments/intermediate-assessment-2017/socio-economics/ (2019).

[81] Thornton, A. et al. *Initial Natural Capital Accounts for the Uk Marine and Coastal Environment*. *Final Report*. Report prepared for the Department for Environment Food and Rural Affairs (2019).

[82] Ajani, J. *Carbon Stock Accounts*. Information Paper for the United Nations Statistics Division Technical Expert Meeting on Ecosystem Accounts, London, 5–7 December (2011). ***This paper examines issues connected with the use of the SEEA for ecosystem carbon accounting, including measurement and valuation of ocean carbon stocks***.

[83] United Nation - Statistical Commission - Report on the fifty-first session (3-6 March 2020), Economic and Social Council, Official Records, 2020 - Supplement No. 4: https://unstats.un.org/unsd/statcom/51st-session/documents/2020-37-FinalReport-E.pdf.

[84] Turner RK, Badura T, Ferrini S (2019). Natural capital accounting perspectives: a pragmatic way forward. Ecosyst. Health Sustainability.

[85] van de Ven, P., Obst, C. & Edens, B. Accounting treatments when integrating ecosystem accounts in the SNA. Paper prepared for SEEA EEA Revision coordinated by the United Nations Statistics Division OSPAR Assessment Portal. *Socioeconomics of the OSPAR Maritime Area – Towards and Assessment Framework*. https://oap.ospar.org/en/ospar-assessments/intermediate-assessment-2017/socio-economics/ (2019).

[86] Acquaye A (2017). Measuring the environmental sustainability performance of global supply chains: A multi-regional input-output analysis for carbon, sulphur oxide and water footprints. J. Environ. Manag..

[87] Brizga J, Feng K, Hubacek K (2017). Household carbon footprints in the Baltic States: a global multi-regional input–output analysis from 1995 to 2011. Appl. Energy.

[88] Bordt M (2018). Discourses in ecosystem accounting: a survey of the expert community. Ecol. Econ..

[89] Hein, L. et al. Progress in natural capital accounting for ecosystems. *Science* **367**, 514–515 (2020). 10.1126/science.aaz8901 32001645

[90] Fuso Nerini F (2019). Connecting climate action with other Sustainable Development Goals. Nat. Sustainability.

[91] Le Blanc, D., Freire, C. & Vierros, M. Mapping the linkages between oceans and other Sustainable Development Goals: a preliminary exploration*. DESA Working Paper* 149 (2017).

[92] Hardin G (1968). The tragedy of the commons. Science.

[93] Brun A (2016). Conference diplomacy: the making of the Paris Agreement. Politics Gov..

[94] Christoff P (2016). The promissory note: COP 21 and the Paris Climate Agreement. Environ. Politics.

[95] Pauw WP (2017). Beyond headline mitigation numbers: we need more transparent and comparable NDCs to achieve the Paris Agreement on climate change. Clim. Change.

[96] Gallo ND, Victor DV, Levin LA (2017). Ocean commitments under the Paris Agreement. Nat. Clim. Change.

[97] United Nations Environment Programme - The Regional Seas Programme: https://www.unenvironment.org/explore-topics/oceans-seas/what-we-do/working-regional-seas/why-does-working-regional-seas-matter.

[98] Duarte CM, Middelburg JJ, Caraco N (2005). Major role of marine vegetation on the oceanic carbon cycle. Biogeosciences.

[99] Najjar RG (2018). Carbon budget of tidal wetlands, estuaries, and shelf waters of eastern North America. Glob. Biogeochemical Cycles.

[100] Mcleod E (2011). A blueprint for blue carbon: toward an improved understanding of the role of vegetated coastal habitats in sequestering CO_2_. Front. Ecol. Environ..

