# Supplementary material for: Climate action requires new accounting guidance and governance frameworks to manage carbon in shelf seas
Source: Nat Commun. 2020 Sep 15;11:4599. doi: 10.1038/s41467-020-18242-w (PMC7492245; doi:10.1038/s41467-020-18242-w)
Supplement: Supplementary file 1 — Peer Review File [file 41467_2020_18242_MOESM1_ESM.docx]

Peer Review Information

**Journal:** Nature Communications

**Manuscript title:** Climate action requires new accounting guidance and governance frameworks to manage carbon in shelf seas

**Corresponding author name(s):** Dr Tiziana Luisetti

**Editorial notes:**

**none**

**Reviewer comments & decisions:**

**Reviewer comments, first version:**

Reviewer #1 (Remarks to the Author):


I found this a thought-provoking paper that raises many questions concerning the basis for, and challenges associated with inclusion, reporting and management of shelf sea sediment carbon stocks as part of national greenhouse gas (GHG) inventories. I think the paper addresses a topical issue of both scientific and broader relevance. It includes appropriate citations spanning the different facets of this topic. Since it is a review or perspective piece, there is no specific scientific data that is reported that requires interpretation. Rather it attempts to highlight underlying processes that require consideration if shelf carbon stocks are to be incorporated as part of GHG reporting protocols. Here are a few points I would like to offer:


1. Not all deposited carbon is created equal with respect to its relevance to GHG sources and sinks. This is particularly so for terrestrial carbon, a good and well-studied example in this regard are the rivers and marginal seas surrounding the island of Taiwan (e.g., see papers by R.G. Hilton and co-authors, and by S.J. Kao and co-authors). This system has several notable characteristics:

(a) Taiwan has some of the highest riverine sediment yields (sediment flux per unit drainage basin area) in the world, and this and other mountainous islands in the Into-Pacific archipelago are considered to account for a significant (up to ~40%) fraction of global terrestrial POC export to the ocean. However, a large proportion of this POC derives from rapid exhumation and erosion of sedimentary rocks that contain organic carbon of geological age. Export and redeposition of this fossil carbon in marine sediments has no net effect on the CO2 inventories (unless it is oxidised along the way, in which case it is a CO2 source). This is very different from sequestration of POC from the terrestrial biosphere, which constitutes a CO2 sink. Therefore, it is important to account not only for the amount of OC accumulating in proximal marine sediments but also its origin.

(b) Taiwan as an active orogen has very narrow shelves (especially on the southern and eastern flanks of the island) of a few km in width, and the shelves are incised by submarine canyons. This means that the majority of exported terrestrial POC (and marine POC produced in overlying waters) is directly injected into and deposited in the deep sea. These sediments may well be within the EEZ but they are definitely at water depths > 200m. The point of the above is not to suggest that Taiwan is discussed as a different end member to the shallow marginal seas of the North West European Shelf (although it certainly offers a sharp contrast to such systems), but to point out that such settings are not considered in the discussion, yet may also be very relevant (e.g., for carbon accounting of a nation like Taiwan).


2. The Northern European shelf sea system is taken as an example to illustrate several points of discussion within the paper. As stated, this system is selected because it is well studied and also because it highlights several of the complex issues surrounding the attribution of carbon stocks due to the number of bordering maritime nations and the large-scale current systems that moves water and particles around. This discussion is at a qualitative level, for example, pointing out the major current trajectories that advect POC towards the Norwegian Trench that serves as a regional sedimentary sink. I think it would be useful to illustrate the importance of the different points highlighted in the text on estimates of C stocks for each of the national marginal sea areas. For example, how would the Great Britain (GB) and Norway (N) marine sediment carbon stocks differ if the estimated fraction of POC that is laterally advected out of the respective EEZ and deposited in the Norwegian Trench is included or excluded? Does it make a big difference, and how might this difference compare with, for example, estimates in the actual carbon stocks stemming different methodological approaches and assumptions?


3. What is known about the timescales of translocation of POC from one shelf region to another? If this occurs via surface currents then I would imagine the transport would be quite fast (months, years), implying that it could impact carbon accounting on short (e.g., inter annual) timescales. However, if the transport occurs at the seafloor in a bottom boundary layer then I expect long-range transport over 10s to 100s of km might be very slow (hundreds or thousands of years?). These two different scenarios would have different implications for assessing [changes in] sediment carbon stocks moving between EEZs.


4. I think the manuscript in its current form is longer than it needs to be and I have the sense the is some repetition and revisiting of similar topics on more than one occasion throughout the paper (albeit from different perspectives). The manuscript covers quite a lot of ground as it seeks to bridge the scientific, economic and socio-political aspects of carbon in shelf seas, however I think it could be more concise. In this regard (although I am not sure what is allowed within the Nature Communications format), I think it could be useful to wrap up the manuscript with concise, bulleted point that summarize the key recommendations that would better inform scientists and policy makers on this topic.


5. Specific points:

- The manuscript uses the term "carbon burial” to describe capture and storage of carbon in soils (line 38). Soils do not bury carbon per se. Unlike sediments which accumulate through deposition, soil develop upwards on a solid substrate and incorporate carbon from overlying biomass. I would avoid using the term carbon burial in this context.

- Reference #18 cited on line 172 does not seem to be the right reference in the reference list.

- Figure 1 would benefit from an additional arrow parallel to the coast indicating along-shelf (instead of across-shelf) transport. This would then directly link to the processes indicated in Figure 2.

- Please check the numbers in Table 1 (which also might benefit from a 3rd column that indicates the global “burial” of organic carbon, e.g., petagrams C yr-1). The table legend states that "on a global scale, the carbon sink in shelf systems is equivalent to that in tropical forests”. Taking the numbers for the table for the area of shelf sediments (2700 x 10^6 ha) and tropical forests (1962 x 10^6 ha) and multiplying these by the corresponding Org C burial fluxes (10^gC ha-1 yr -1), I calculate a global annual burial flux in shelf sediments and tropical forests of ~ 0.46 x10^15 gC yr-1 (which seems a bit high) and 0.078 x 10^15 gC yr-1, respectively. This implies that shelf sediments “bury” > 5x more C than tropical forests, and are thus not “equivalent". Also, the legend suggests that a range of values for shelf sediments should be indicated depending on the % of muddy (as opposed to sandy) sediment cover, but no such range is shown in the table. Finally, the origin of the (and the type of) data that is used to derive the calculations for shelf sediment OC should be indicated.

- Line 293: "damages estimated in the range of billions” - billions of what? GBP or dollars?


Reviewer #2 (Remarks to the Author):

The article is well-written and discusses a complex and urgent problem, that is climate action as it relates to the governance and management of carbon in shelf seas. The authors put forward original arguments, which are well presented and therefore, makes a relevant contribution to the academic literature and policy. Using the North Sea example, the authors answered the critical questions set out in the introduction and also expounded on the implications of their findings for the existing carbon assessment and management framework, while also making recommendations on how to avert any impending tragedy of the commons.

I have one recommendation: there appears to be a missed opportunity to address what the current situation means for the attainment of the relevant SDGs – for example SDGs 13 and 14 aimed at combating climate change and life underwater, respectively. Inference to the SDGs in the introduction indicates an awareness of the interconnectedness of the issues discussed and the goals; the authors should, therefore, consider adding some sentences to address the implications to the relevant SDGs of not embracing new accounting guidance and frameworks to manage the carbon in shelf seas.

**Author rebuttal, first version:**

|  | **Comment** | **Reply** |
| --- | --- | --- |
| **Reviewer** **1** | I found this a thought-provoking paper that raises many questions concerning the basis for, and challenges associated with inclusion, reporting and management of shelf sea sediment carbon stocks as part of national greenhouse gas (GHG) inventories. I think the paper addresses a topical issue of both scientific and broader relevance. It includes appropriate citations spanning the different facets of this topic. Since it is a review or perspective piece, there is no specific scientific data that is reported that requires interpretation. Rather it attempts to highlight underlying processes that require consideration if shelf carbon stocks are to be incorporated as part of GHG reporting protocols. | We thank the reviewer for their comment and encouragement. |
|  | Not all deposited carbon is created equal with respect to its relevance to GHG sources and sinks. This is particularly so for terrestrial carbon, a good and well-studied example in this regard are the rivers and marginal seas surrounding the island of Taiwan (e.g., see papers by R.G. Hilton and co-authors, and by S.J. Kao and co-authors). |  |
|  | (a) Taiwan has some of the highest riverine sediment yields (sediment flux per unit drainage basin area) in the world, and this and other mountainous islands in the Into-Pacific archipelago are considered to account for a significant (up to ~40%) fraction of global terrestrial POC export to the ocean. However, a large proportion of this POC derives from rapid exhumation and erosion of sedimentary rocks that contain organic carbon of geological age. Export and redeposition of this fossil carbon in marine sediments has no net effect on the CO2 inventories (unless it is oxidised along the way, in which case it is a CO2 source). This is very different from sequestration of POC from the terrestrial biosphere, which constitutes a CO2 sink. **Therefore, it is important to account not only for the amount of OC accumulating in proximal marine sediments but also its origin.** | We agree completely with the reviewer’s general point that it is important to understand the origin of the deposited carbon. We also agree that in different geological settings the behaviour of carbon on the shelf will be different and in convergent plate margin setting such as Taiwan an important amount of organic carbon will be deposited beyond the shelf. We did allude to these points in the original text but take this opportunity to make the point more clearly and also to cite the Taiwanese examples because they do provide a valuable contrast to the North Sea (a passive geological margin setting). This gives us the opportunity to emphasise that the storage of organic carbon is a global feature but that the site and nature of that storage is different in geographic settings. Hence, as we illustrate with our North Sea example, the management of these shelf carbon stocks requires both an understanding of the natural science of the carbon deposition and also a socio-political understanding of the regional shelf system.  We have added a new paragraph in the previous line 176.  We also agree with the reviewers point about the importance of distinguishing between terrestrial fossil carbon. The text already does note the need to understand the origins of the carbon and the relative importance of fluvial and marine sources (e.g. previous lines 135-7 and 349), but we take the opportunity to strengthen this point with additional text in the previous line 349. |
|  | (b) Taiwan as an active orogen has very narrow shelves (especially on the southern and eastern flanks of the island) of a few km in width, and the shelves are incised by submarine canyons. This means that the majority of exported terrestrial POC (and marine POC produced in overlying waters) is directly injected into and deposited in the deep sea. These sediments may well be within the EEZ but they are definitely at water depths > 200m. **The point of the above is not to suggest that Taiwan is discussed as a different end member to the shallow marginal seas of the North West European Shelf (although it certainly offers a sharp contrast to such systems), but to point out that such settings are not considered in the discussion, yet may also be very relevant (e.g., for carbon accounting of a nation like Taiwan).** | In line with the previous reply to comment (a) we have added some text in the previous line 176. |
|  | 2. The Northern European shelf sea system is taken as an example to illustrate several points of discussion within the paper. As stated, this system is selected because it is well studied and also because it highlights several of the complex issues surrounding the attribution of carbon stocks due to the number of bordering maritime nations and the large-scale current systems that moves water and particles around. This discussion is at a qualitative level, for example, pointing out the major current trajectories that advect POC towards the Norwegian Trench that serves as a regional sedimentary sink. **I think it would be useful to illustrate the importance of the different points highlighted in the text on estimates of C stocks for each of the national marginal sea areas.** For example, **how would the Great Britain (GB) and Norway (N) marine sediment carbon stocks differ if the estimated fraction of POC that is laterally advected out of the respective EEZ and deposited in the Norwegian Trench is included or excluded?** **Does it make a big difference, and how might this difference compare with, for example, estimates in the actual carbon stocks stemming different methodological approaches and assumptions?** | Some of the authors of this paper have also been involved in a recent carbon budget for the North Sea (Legge et al 2020) which does include the information the reviewer suggests we include. This reference is now in the reference list.  The Norwegian trench stores approximately 10% of the organic carbon stock within the North Sea and we now cite the Legge et al paper (in the previous Lines: 93, 172 and 315 and in line 156, in the caption to Figure 2). |
|  | 3. **What is known about the timescales of translocation of POC from one shelf region to another?** If this occurs via surface currents then I would imagine the transport would be quite fast (months, years), implying that it could impact carbon accounting on short (e.g., inter annual) timescales. However, if the transport occurs at the seafloor in a bottom boundary layer then I expect long-range transport over 10s to 100s of km might be very slow (hundreds or thousands of years?). These two different scenarios would have different implications for assessing [changes in] sediment carbon stocks moving between EEZs. | We thank the reviewer for giving us the chance to include these considerations in our manuscript. We have inserted a new paragraph in previously line 147 just after (Figure 2) and before ‘We focus’. |
|  | 4. I think **the manuscript in its current form is longer than it needs to be** and I have the sense the is some repetition and revisiting of similar topics on more than one occasion throughout the paper (albeit from different perspectives). The manuscript covers quite a lot of ground as it seeks to bridge the scientific, economic and socio-political aspects of carbon in shelf seas, however I think it **could be more concise**. In this regard (although I am not sure what is allowed within the Nature Communications format), I think it could be useful to **wrap up the manuscript with concise, bulleted point that summarize the key recommendations that would better inform scientists and policy makers on this topic.** | We thank the reviewer for pointing this out and giving us the chance to sharpen our message whilst accommodating the insertion of new text as required. We have made some changes, including wrapping up the manuscript more concisely with bullet points in our conclusions.  Details as follow (based on the page numbering of the previous PDF):  Line 71-73: removed  Line 82: removed  Line 83: moved  Line 85-86: removed  Line 97: sentence removed  Line 102 and 104: one sentence removed and the rest a little re-phrased  Line 147: inserted new paragraph  Line 176: inserted new paragraph  Line 185-188: words in brackets removed  Line 293-295: removed  Line 313-315: removed  Line 320: paragraph from lines 343-346 moved here  Line 324-326: removed  Line 335-338: reworded  Line 349: new sentence added  Line 377-379: words removed  Line 419-423: rephrased to incorporate an update  Line 424: text from lines 437-439 moved here  Line 427: text from lines 505-508 moved here  Line 436-437: removed  Line 440: new text inserted here  Line 445: text from line 496 inserted here  Line 450-454: rephrased  Line 458-459: words removed  Line 461: words added  Line 464: removed  Line 479-481: removed  Line 485-487: removed  Line 489-492: removed  Line 496: text from lines 508-513 moved here  Line 496-498: moved to Line 445  Line 498-501: removed  Line 502-505: removed  Line: 505-508: moved in line 427.  We have also included more references as appropriate. |
|  | The manuscript uses the term "**carbon burial**” to describe capture and storage of carbon in soils (**line 38**). Soils do not bury carbon per se. Unlike sediments which accumulate through deposition, soil develop upwards on a solid substrate and incorporate carbon from overlying biomass. I would avoid using the term carbon burial in this context. | We thank the reviewer for highlighting the current confusion between the definition of sediments and soils and the processes that lead to their formation. The term soil is used in this document to describe the substrata in coastal environments vegetated by mangrove forests, tidal marshes and seagrass beds, which is in-line with pedologists that define soils as sediments supporting emergent vegetation. The coastal vegetated soils have been accumulating, in some cases, over millennia, and the soil horizon has been rising in pace with sea level rise. Due to the water-logged and anoxic nature of these soils, the continuous accumulation of carbon results in its burial. While we agree that the term ‘carbon burial’ should be removed from line 38, in the context of terrestrial soil, we have retained the term in the context of mangrove, tidal marsh and seagrass soils and shelf sediments. |
|  | **Reference #18 cited on line 172** does not seem to be the right reference in the reference list. | We thank the reviewer for spotting this.    We have replaced the reference previously in #18 with the following:  Wilson, R.J., Speirs, D.C., Sabatino, A., and M.R. Heath. A synthetic map of the north-west European Shelf sedimentary environment for applications in marine science. *Earth Syst. Sci. Data* 10, 109-130 (2018). doi.org/10.5194/essd-10-109-2018 |
|  | **Figure 1 would benefit from an additional arrow parallel to the coast indicating along-shelf** (instead of across-shelf) transport. This would then directly link to the processes indicated in Figure 2. | We thank the reviewer for giving us the chance to improve our infographics. We have now modified Figure 1 as suggested. |
|  | Please **check the numbers in Table 1** **(which also might benefit from a 3rd column that indicates the global “burial” of organic carbon, e.g., petagrams C yr-1)**. The table legend states that "**on a global scale, the carbon sink in shelf systems is equivalent to that in tropical forests**”. Taking the numbers for the table for the area of shelf sediments (2700 x 10^6 ha) and tropical forests (1962 x 10^6 ha) and multiplying these by the corresponding Org C burial fluxes (10^gC ha-1 yr -1), I calculate a global annual burial flux in shelf sediments and tropical forests of ~ 0.46 x10^15 gC yr-1 (which seems a bit high) and 0.078 x 10^15 gC yr-1, respectively. **This implies that shelf sediments “bury” > 5x more C than tropical forests, and are thus not “equivalent".** Also, the legend suggests that **a range of values for shelf sediments should be indicated depending on the % of muddy (as opposed to sandy) sediment cover, but no such range is shown in the table.** Finally, **the origin of the (and the type of) data that is used to derive the calculations for shelf sediment OC should be indicated.** | We thank the reviewer for giving us the chance to make our Table 1 more accessible and precise.  We have checked the numbers in Table 1 and added a third column as suggested – estimates are reported in Tg yr^-1.  We have substituted the word ‘equivalent’ with ‘comparable’, because we want to highlight both the uncertainty in the measurements of the fluxes and that the shelf sea storage number is probably large and important. So, we have also added a range of values (details are found in the foot note of Table 1: The depositional areas on the shelf varies with their geographic setting. The range given for global C burial represents estimates for a depositional areal extent of muds between 10%^96^ to 30%^97^of the total shelf area reported in column two). |
|  | **Line 293**: "damages estimated in the range of billions” - billions of what? GBP or dollars? | We thank the reviewer for spotting this. We have now added the currency (US dollars). |
| **Reviewer 2** | The article is well-written and discusses a complex and urgent problem, that is climate action as it relates to the governance and management of carbon in shelf seas. The authors put forward original arguments, which are well presented and therefore, makes a relevant contribution to the academic literature and policy. Using the North Sea example, the authors answered the critical questions set out in the introduction and also expounded on the implications of their findings for the existing carbon assessment and management framework, while also making recommendations on how to avert any impending tragedy of the commons. | We thank the reviewer for this positive comment and their encouragement. |
|  | There appears to be a missed opportunity to **address what the current situation means for the attainment of the relevant SDGs** – for example **SDGs 13 and 14 aimed at combating climate change and life underwater, respectively**. Inference to the SDGs in the introduction indicates an awareness of the interconnectedness of the issues discussed and the goals; the authors should, therefore, consider **adding some sentences to address the implications to the relevant SDGs of not embracing new accounting guidance and frameworks to manage the carbon in shelf seas**. | We thank the reviewer for giving us the chance to say something more about the SDGs. We have added a new paragraph in the previous line 436 (some text there has been removed and/or moved in other paragraph to reduce the length of the manuscript as required by reviewer 1 – please, see details in the replied to reviewer 1 section). |

**Reviewer comments, second version:**

Reviewer #1 (Remarks to the Author):

I think the revised manuscript is significantly improved and I would recommend publication. The manuscript is well written, addresses an important and timely topic in a balanced fashion and from a broad range of different perspectives. My comments on a prior version of this manuscript have been satisfactorily addressed. I have just a few very minor points:

Line 228 "Emissions to and removals from the atmosphere of CO2 by coastal wetlands”: This could be rephrased to flow more smoothly.

Line 421-422: "Marine data are not usually available from a set of centralised databases, making them difficult to find and use” This is not entirely true. For example, Pangaea (https://www.pangaea.de/) is a broadly used and extensive repository of marine data of the type necessary to determine marine sediment carbon stocks. The issue is that it is not necessarily an easy task to extract this information in a facile manner.

There is a recent paper (Atwood T.B. et al 2020 Global patterns in marine sediment carbon stocks. Frontiers in Marine Science 7:165) that is relevant to the present study and could be cited. In addition to providing a revised assessment of C stocks, it employs use of machine learning tools to derive these estimates. This brings up an issue of the enormous logistical challenges of actually quantifying stocks (e.g., through oceanographic cruises and analysis of retrieved sediment cores) in the world’s shelf seas (which perhaps could be better highlighted). Machine learning and similar approaches are one way to try to address data gaps.

The impacts of bottom trawling of sediment carbon stocks is briefly mentioned. There are several recent papers on the impact of bottom trawling on sediment displacement and carbon contents. It might be useful to cite one or two of these as specific examples (e.g., Pusceddu et al 2014 PNAS 111 8861-8866; Paradis S. et al 2019 Biogeosciences 16 4307-4320).

Reviewer #2 (Remarks to the Author):

Thank you for your revisions on the manuscript.

I am glad my comments were useful and thanks for addressing it.
The paper makes a vital contribution to the field, and I am delighted to recommend it for publication.

**Author rebuttal, second version:**

|  | **Comment** | **Reply** |
| --- | --- | --- |
| **Reviewer** **1** | I think the revised manuscript is significantly improved and I would recommend publication. The manuscript is well written, addresses an important and timely topic in a balanced fashion and from a broad range of different perspectives. My comments on a prior version of this manuscript have been satisfactorily addressed. I have just a few very minor points: | We thank the reviewer for this comment and for their suggestions, which allowed us to improve our manuscript. |
|  | Line 228 "Emissions to and removals from the atmosphere of CO2 by coastal wetlands”: This could be rephrased to flow more smoothly. | Line 228 has been rephrased with: The storage and loss of atmospheric CO_2_ from coastal wetlands has multiple causes. |
|  | Line 421-422: "Marine data are not usually available from a set of centralised databases, making them difficult to find and use” This is not entirely true. For example, Pangaea ([https://www.pangaea.de/](https://eur02.safelinks.protection.outlook.com/?url=https%3A%2F%2Fwww.pangaea.de%2F&data=02%7C01%7C%7Ca9e8275bbab1407f6ad208d828cb85a3%7Ceeea3199afa041ebbbf2f6e42c3da7cf%7C0%7C0%7C637304201895273253&sdata=0ZvjzmqG4uO0lctC5Kl%2FkhbQyC%2BHlPGRW349EyqDSeo%3D&reserved=0)) is a broadly used and extensive repository of marine data of the type necessary to determine marine sediment carbon stocks. The issue is that it is not necessarily an easy task to extract this information in a facile manner. | We thank the reviewer for highlighting this to us. We have rephrased Line 421-422 as: Marine data are not always readily available in central databases, and it is therefore sometimes difficult to extract required information. |
|  | There is a recent paper (Atwood T.B. et al 2020 Global patterns in marine sediment carbon stocks. Frontiers in Marine Science 7:165) that is relevant to the present study and could be cited. In addition to providing a revised assessment of C stocks, it employs use of machine learning tools to derive these estimates. This brings up an issue of the enormous logistical challenges of actually quantifying stocks (e.g., through oceanographic cruises and analysis of retrieved sediment cores) in the world’s shelf seas (which perhaps could be better highlighted). Machine learning and similar approaches are one way to try to address data gaps. | We thank the reviewer for mentioning this nice and useful paper to us. We have therefore added the reference to this valuable new comprehensive geochemical evaluation of marine carbon stores in Lines 104 and 186. |
|  | The impacts of bottom trawling of sediment carbon stocks is briefly mentioned. There are several recent papers on the impact of bottom trawling on sediment displacement and carbon contents. It might be useful to cite one or two of these as specific examples (e.g., Pusceddu et al 2014 PNAS 111 8861-8866; Paradis S. et al 2019 Biogeosciences 16 4307-4320). | We thank the reviewer for mentioning these nice examples of the impacts of trawling, albeit in rather deeper settings than those we consider. We have now added the references to Pusceddu et al and Paradis et al in Line 201. |
| **Reviewer 2** | Thank you for your revisions on the manuscript.  I am glad my comments were useful and thanks for addressing it. The paper makes a vital contribution to the field, and I am delighted to recommend it for publication. | We thank the reviewer for this comment and for their suggestions, which allowed us to improve our manuscript. |

**Reviewer comments, third version:**

**Author rebuttal, third version:**
